# Expression of *HOXA10* and *HOXA11* in the endometrium of infertile patients with chronic endometritis

**DOI:** 10.5935/1518-0557.20240035

**Published:** 2024

**Authors:** Ana Carolina Mendonça Hissa de Sá, Luiz Felipe Bittencourt de Araujo, Mylena Zuim Sanson, Tamyres Souza Garcia Alvim Ranzato, Amanda Rezende Passarelli Tostes, Ivan Andrade de Araujo Penna

**Affiliations:** 1 Universidade Federal Fluminense, Departamento de Saúde Materno-Infantil, Niterói, Rio de Janeiro, Brazil; 2 Medical student. Universidade UNIGRANRIO - RJ, Brazil

**Keywords:** infertility, chronic endometritis, immunohistochemistry, HOXA10, HOXA11

## Abstract

**Objective:**

The study aimed to evaluate the impact of CE on the expression of *HOXA10* and *HOXA11* during the late proliferative phase in the endometrium of infertile women.

**Methods:**

A prospective, translational cohort study was conducted in partnership with the Hospital Universitário Antônio Pedro in Niterói and the Clínica Ginendo in Rio de Janeiro after approval by the Ethics Committee. The patients were selected to participate in the study after showing an indication for hysteroscopy. All participants were divided into three groups: infertile women with endometritis (n=10), infertile women without endometritis (n=17) and fertile women without endometritis (n=10). At hysteroscopy, two endometrial samples were obtaneid, with one sent for histopathological examination per the gynecologist’s request and the other used for immunohistochemistry procedures to evaluate the expression of *CD138, HOXA10* and *HOXA11. CD138* was used to confirm the diagnosis of CE. The analysis of *HOXA10* and *HOXA11* was performed using the HScoring method for immunohistochemistry with polyclonal antibodies.

**Results:**

Women with and without endometritis had lower *HOXA10* and *HOXA11* expression values than women in the control group (fertile women without endometritis).

**Conclusions:**

The expression of *HOXA10* and *HOXA11* during the proliferative phase is not significantly different between infertile women with endometritis and infertile women without endometritis. Translational studies with a larger number of patients should be performed.

## INTRODUCTION

Despite advances in diagnostic methods, 15% of infertility causes remain unknown (Szczepańska *et al*., 2011). The interaction between the endometrium and the embryo, as well as the receptivity of the endometrium is responsible for approximately 60% of implantation failures (Riyanti *et al*., 2020). Human embryonic implantation is a complex process that requires fine synchronization between the embryo and a receptive endometrium, as well as an intricate molecular dialog between the two (Mahajan, 2015). Such events occur in a receptive endometrium stimulated by ovarian steroid hormones, namely, estrogen and progesterone, to provide an anatomical site for embryo implantation (Koot & Macklon, 2013).

Among the histomorphological criteria for embryonic implantation to occur, decidualization of the stroma and development of pinopods and microvilli of the luminal epithelium are required (Dunn *et al*., 2003). At the molecular level, alterations in the gene expression of cytokines and growth factors and the transcription of adhesion molecules, such as *HOX* genes, are involved (Kalma *et al*., 2009; Paria *et al*., 2002).

Throughout the menstrual cycle, the cells of the uterine endometrium constantly undergo proliferation and differentiation processes that are analogous to many changes in embryonic development (Bagot *et al*., 2001). Sex steroids dictate the pattern of *HOX* gene expression in the adult endometrium, playing a key role in embryo implantation (Taylor, 2000). *HOXA10* and *HOXA11* are expressed in the endometrial glands and stroma throughout the menstrual cycle. In the endometrial glands, these genes have peak expression during the secretory phase and play a crucial role in the success of implantation. In endometrial stroma, gene expression is constant and invariable at all stages of the menstrual cycle (Taylor, 2000; Sarno *et al*., 2005). Women with a high implantation rate have high expression of *HOXA10* and *HOXA11* in the secretory phase, suggesting that maternal expression of these genes is essential for successful implantation (Du & Taylor, 2015).

Chronic endometritis is defined as chronic inflammation of the endometrium. It is associated with an increased prevalence of recurrent pregnancy loss and implantation failure after in vitro fertilization (Zargar *et al*., 2020; Goldberg *et al*., 2019; Johnston-MacAnanny *et al*., 2010; Cicinelli *et al*., 2005). Chronic endometritis lesions are often associated with pelvic inflammatory diseases and intermenstrual bleeding (Smith *et al*., 2010). Pathophysiologically, it is characterized by the presence of plasma cells in the endometrium, especially in the stroma (Nucci & Oliva, 2009). Diagnosis is usually based on endometrial biopsy based on hysteroscopy, endometrial stromal plasma cell count and immunohistochemical staining for CD138 (Kitaya & Yasuo, 2011; Kasius *et al*., 2011). The possible relationship between chronic endometritis, infertility and perinatal complications has recently been reported in the literature (Kitaya *et al*., 2016). Studies indicate that even after treatment with antibiotics, patients with chronic endometritis continue to have low implantation rates, suggesting that the pathology may cause endometrial anomalies other than those generated by microorganisms that lead to tissue inflammation (Johnston-MacAnanny *et al*., 2010). The molecular mechanisms that may promote implantation failure in these patients have not yet been fully elucidated (Kitaya *et al*., 2016). Therefore, in this study we attempted to demonstrate a possible impact of chronic endometritis on the expression of HOXA10 and HOXA11 in infertile women.

## MATERIAL AND METHODS

A prospective, translational cohort study was conducted in partnership with the Antônio Pedro University Hospital in Niterói, the Ginendo Clinic in Rio de Janeiro. Cellular Interactions Laboratory of the Institute of Biomedical Sciences at UFRJ for immunohistochemical processing. CD138, *HOXA10* and HOXA11 after approval was obtained from the Research Ethics Committee of Fluminense Federal University. The patients were selected and invited to participate in the study after showing an indication for hysteroscopy due to infertility in the couple. The invitation was made before the examination without any impairment to the treatment or follow-up of the patient.

The material for biopsy was removed, and part of it was targeted for research. All participants signed informed consent forms. The patients who participated in the study were divided into three groups: women with endometritis and infertility women; women without endometritis but with infertility; and women without endometritis and infertility. Infertility was defined as the inability to conceive after 12 months for women under the age of 35 years or after 6 months for women over 35 years of age. The division of the endometritis groups occurred after hysteroscopy with biopsy and confirmation with positive CD138.

The inclusion criteria for the 3 groups were 1) aged between 30 and 40 years; 2) performing hysteroscopy during the same period of the menstrual cycle, between the 8th and 12th day of the cycle, with the dates of the cycle confirmed by the patient’s history; 3) fertile women, defined as women with previous pregnancy and childbirth; and 4) indication for hysteroscopy due to infertility if the woman had endometritis and infertility. The exclusion criteria for the endometritis and infertility group were as follows: 1- polycystic ovary syndrome/anovulation; 2- premature ovarian failure; 3- hyperprolactinemia; 4- hyper/hypothyroidism; 5- tubal obstruction; 6- history of endometriosis; 7- previous surgery to remove cervical cancer; 8- endometrial hyper/hypoplasia; 9- uterine synechiae; 10- uterine malformation; 11- signs of vaginosis, such as those caused by *Streptococcus agalactiae, Candida* spp., *Gardenerella vaginalis, Trichomonas* spp., *Mycoplasma genitalium, Chlamydia trachomatis, Neisseria gonorrhea*, or *Mycoplasma hominis*; 12- BMI > 40 kg/m^2^; 13- previous endometrial ablation; 14- anterior embolization of the uterine artery; 15- presence of submucosal myoma or myoma that distorts the cavity on hysteroscopy; 16- presence of type 4 myoma with (intramural) mean diameter > 4 cm; 17- presence of polyps; 18- presence of hydrosalpinx on hysterosalpingography or ultrasound; 19- suspected clinical signs of gonorrhea or chlamydia; or 20- chronic use of glucocorticoids (except nasal preparations).

Two samples of approximately 1 mm in size were taken from each patient during the procedure in all groups: one sample was sent for histopathological examination, as suggested by the gynecologist and the other sample was used for the immunohistochemistry procedures of the present study.

The samples destined for immunohistochemistry for CD138, *HOXA10* and *HOXA11* staining were subjected to the following procedure. 1- After fixation in 10% buffered formalin for a maximum period of 24 hours, the fragments were dehydrated and clarified. 2- Each cassette was immersed for a total of 45 minutes in 70% alcohol, 80% alcohol, 90% alcohol, 100% absolute alcohol, followed by Xylene I, Xylyl III. 3- The pieces were then subjected to paraffin baths, namely, paraffin I and paraffin II (cleaner), and kept in an oven at 58°C to 62°C for 45 minutes each. 4- Then, the segments were included in metal molds with liquid paraffin for blocking. 5- The tissues were cut into 5 µm-thick sections in a microtome (Semiautomatic Sleeve, NiederOlm), followed by immersion in a water bath at 50°C with distilled water for distension. Then, they were placed in salinized slides and placed in an oven for 20 minutes between 58 and 62°C.

The CD138, *HOXA10* and *HOXA11* immunohistochemical protocol was used, and the reagents were applied with the aid of a pipette. The slides were placed in a humidified chamber under the surface to allow the flow of the reagents.

The slides were deparaffinized following sequential xylene baths, rehydrated in decreasing concentrations of alcohol and washed for 5 minutes in running water. Then, antigenic retrieval was performed by incubating the sections in Trilogy buffer (Cell Marque, Rocklin) at 98°C for 20 minutes in a water bath. Then, to block nonspecific binding (endogenous peroxidase) the sections were incubated with “Peroxidase Block” reagent (Leica, São Paulo) for 5 minutes at room temperature, followed by two washes of 5 minutes each with PBS solution. Soon after, the sections were incubated with primary antibodies against *HOXA10, HOXA11* and CD138 (Thermo Fisher, Waltham) diluted 1:25, 1:50 and 1:25, respectively, for 1 hour at room temperature. Then, the slides were washed twice for 5 minutes with PBS solution.

The development reaction for the detection of cells labeled with CD138, *HOXA10* and *HOXA11* was developed using the “Novolink Polymer Detection System” kit (Leica, São Paulo) according to the manufacturer’s recommendations. In the case of CD138, after incubation with the primary antibody, the sections were incubated with the “Post Primary” reagent (Leica, São Paulo), equivalent to the secondary antibody for amplification of the first signal in antibodies produced in mice, for 30 minutes at room temperature, followed by two 5 minutes washes each with PBS.

For *HOXA11*, the sections were incubated using “Novolink Polymer” (Leica, São Paulo), corresponding to the secondary antibody that binds to antibodies produced in rabbits, for 30 minutes at room temperature, followed by two more washes with PBS for 5 minutes each. For *HOXA10*, the sections were incubated using “Novolink Polymer” (Leica, São Paulo), corresponding to the secondary antibody that binds to antibodies produced in goats, for 30 minutes at room temperature, followed by two more 5 minutes washes with PBS.

The chromogen substrate used in the development reaction of the 3 markers was diaminobenzidine (DAB) associated with hydrogen peroxide (Leica, São Paulo), with a dilution of 50 mL of DAB in 1mL of dilution buffer, which was added to the tissue for 2 to 5 minutes. Positive staining in cells was indicated by brown staining. Counterstaining was performed with hematoxylin (Leica, São Paulo) for 5 minutes.

For the diagnosis of chronic endometritis, the samples were selected by hysteroscopy and stained with antibodies against CD138 (plasma cell transmembrane protein) and the number of plasma cells was quantified. Samples with five or more plasma cells labeled by immunohistochemistry were considered positive using 20 high-power fields (most selective criterion) (Johnston-MacAnanny *et al*., 2010; Xu *et al*., 2020; Kimura *et al*., 2019; Bouet *et al*., 2016; McQueen *et al*., 2014).

The analysis of *HOXA10* and *HOXA11* was performed using the HScore technique of immunohistochemistry with polyclonal antibodies. Anti-HOXA10 and anti-HOXA11 primary antibodies conjugated with secondary antibodies that provided the characteristic color of the reaction were used. The technique followed the recommendations of the manufacturer (Thermo Fisher, Waltham).

**Figure 1:**
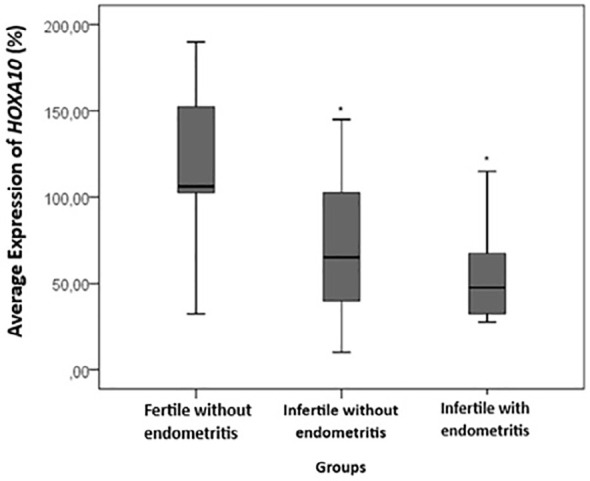
Average percentage of HOXA10 expression in the proliferative phase of the menstrual cycle (8^th^ to 12^th^ day) between the groups: fertile without endometritis, infertile without endometritis and infertile with endometritis. This represents a significant difference compared to the control group (fertile without endometritis) (*p* value<0.05; ANOVA with Tukey’s post hoc test).

**Figure 2:**
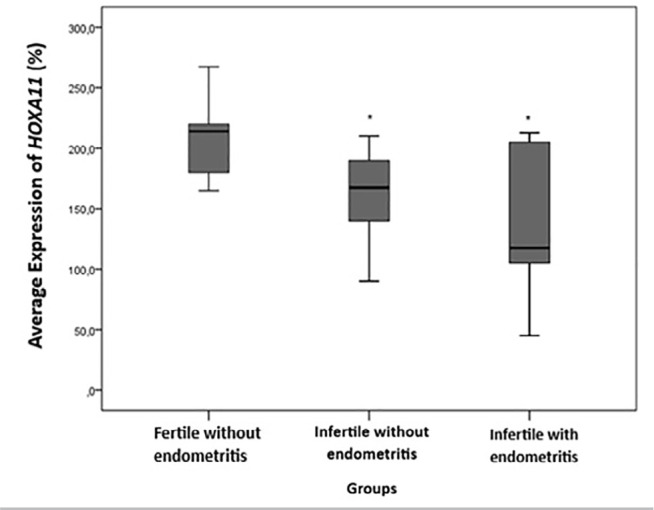
Average percentage of HOXA11 expression in the proliferative phase of the menstrual cycle (8^th^ to 12^th^ day) between the groups: fertile without endometritis, infertile without endometritis and infertile with endometritis. This represents a significant difference compared to the control group (fertile without endometritis). (*p* value<0.05; ANOVA test with Tukey’s post-hoc test).

The HScore technique is a semiquantitative method that correlates the number of stained cells and the intensity of intracellular staining at 40X magnification under a light microscope (Lessey *et al*., 1988; Budwit-Novotny *et al*., 1986). The intensity of staining was classified as undetectable (0), weak but detectable (1), distinct (2) or very strong (3) (Figures 1 and 2). The score was calculated with the formula H = Σ P x i, where P is the percentage of stained cells that were labeled by the antibody and is the intensity score (0, 1, 2 or 3). The slides were analyzed individually by two different blinded observers, and the percentage of labeled cells was recorded with HScore. Statistical analysis of the HScore was performed by ANOVA with Tukey’s post hoc test using a significance level of 0.05.

## RESULTS

The sociodemographic data regarding age, time to pregnancy and number of children were compared to assess the homogeneity of the sample. For this purpose, normality was assessed using the Kolmogorov-Smirnov test, and if the data followed a normal distribution, the comparison was performed using ANOVA with Tukey’s post hoc test. Otherwise, the Kruskal-Wallis test with Dunn’s post hoc test was used. All data were evaluated using SPSS statistical software, version 20.0, and the significance level used was 0.05.

The groups were homogeneous in their initial characteristics in relation to their ages (Table 1). However, the time to pregnancy was longer in infertile group than in the control group, and the number of children was lower in the infertile group than in the control group. It is worth noting that all patients diagnosed with EC had CD138 positivity in more than five cells at 20x magnification.

**Table 1:** Demographic data of the fertile women without endometritis, infertile women without endometritis and infertile women with endometritis.

	Fertile Without endometritis (n=10)	Infertile without endometritis (n=17)	Infertile women With endometritis (n=10)	*p* value
Age (years)	36.00±4.27	37.88±2.64	37.40±4.09	0.415
Time to get pregnant (years)	0.00±0.00	3.47±2.37^*^	3.30±2.11^*^	<0.0001
Number of children	1.90±1.20	0.06±0.24^*^	0.00±0.00^*^	<0.0001

The demographic data were taken from questionnaires answered by the patients before the hysteroscopy. The data was measured in years; the time for pregnancy in the fertile group was smaller than 1 year.

Some of the patients distributed in the infertile groups had already had children before the present study. This number of children was put on the table as demographic data, at the time of the study they were classified as infertile (secondary infertility) since they were attempting to conceive for more than 12 months with no success being younger than 35 years old or 6 months in women being older than 35 years old.

Figure 1 shows the comparison of de *HOXA10* averages according to the evaluated groups in the proliferative phase of the menstrual cycle (8th to 12th day). There was a significant difference between the groups, with infertile women with endometritis having lower *HOXA10* expression values than the women in the control group (fertile without endometritis). This was also observed in the infertile women without endometritis compared to the control group (*p* value=0.003; fertile without endometritis: 116.75±46.31; infertile without endometritis: 71.32±41.04; infertile with endometritis: 54.00±27.03). Although there was no significant difference between infertile women without endometritis and infertile women with endometritis, a reduction in *HOXA10* expression was observed in infertile women with endometritis (-24.3%).

The comparison of the means of *HOXA11* according to the evaluated groups is shown in Figure 2. There was a significant difference between the groups, with infertile women with endometritis having lower *HOXA11* expression values than women in the control group (fertile without endometritis), as was observed for infertile women without endometritis compared to the control group (*p* value=0.001; fertile without endometritis: 212.80±32.99; infertile without endometritis: 159.32±36.16; infertile with endometritis: 137.25±55.87). Although there was no significant difference between infertile women without endometritis and infertile women with endometritis, a reduction in *HOXA11* expression was observed in infertile women with endometritis (-13.8%).

## DISCUSSION

Based on the HScore analysis by immunohistochemistry, it was possible to observe a reduction in the expression of *HOXA10* and *HOXA11* in infertile women compared to women in the control group (fertile without endometritis). These data have already been investigated by Taylor *et al*., 1998, and according to these authors, a few markers are indispensable for implantation, with *HOXA10* being the most important. In this sense, the low expression of *HOXA10* and *HOXA11* leads to abnormal uterine development and impairment of implantation. A difference between that study and the present study is that for ethical reasons, endometrial biopsy was performed in the proliferative phase of the menstrual cycle, unlike previous studies in which this was performed in the secretory phase where *HOXA10* could probably also be expressed.

In contrast to what was found in the present study and that of Taylor *et al*., 1998, the study conducted by Szczepańska *et al*., 2011 in patients with idiopathic infertility, showed that there was no difference between the level of HOXA10 or HOXA11 expression between the infertility and control groups (Szczepańska *et al*., 2011).

There was no significant difference in the expression of *HOXA10* and *HOXA11* between infertile patients with endometritis and those without endometritis (Marin *et al*., 2022). In the present study, a decrease in the expression of both genes was observed, but this result was not significant. This may have occurred due to the number of patients evaluated, the use of a single analytical method (immunohistochemistry) or the period of the cycle in which the endometrial collection was performed. However, it is worth noting that the power of the test was 88% and certifies the significance of the study result.

A recent study with a similar sample size (n=13 with chronic endometritis and n=16 without chronic endometritis) that evaluated the expression of *HOXA10* and *HOXA11* in patients with and without endometritis also found similar data, namely, that there was no significant difference between groups (Marin *et al*., 2022). There is a significant difference between the present study and that performed by (Marin *et al*., 2022), because in the present study, a control group of fertile patients without endometritis was created, that is, a negative control that, from the methodological point of view, reduces the chances of error. In the current study, immunohistochemistry and semiquantitative analysis were performed using the HScore to determine the percentage of *HOXA10* and *HOXA11* expression.

Our analysis was at the protein level, and we did not have an evaluation methodology at the molecular level, for example quantitative techniques, such as polymerase chain reaction (PCR). If this second technique was used, which was not possible for structural reasons, we might also be able to find a difference in relation to our study. However (Marin *et al*., 2022), although they used PCR, they also found no significant difference, showing that eventually a new molecular level of analysis would also corroborate our results. Due to issues imposed by the Ethics Committee so that there was no harm to the patients who participated in the study, the period of hysteroscopy performance was in the late follicular phase, which may have impacted the HScore evaluations since the best result would be in the secretory phase.

In normal fertile women, the expression of HOXA10 and HOXA11 significantly increases during the luteal phase, with the peak occurring during the implantation window, and remains at an elevated level until the end of the cycle (Du & Taylor, 2015). Other studies concluded that this difference in expression during the menstrual cycle occurs only in the region of the endometrial glandular epithelium and not in the stromal compartment (Sarno *et al*., 2005). Taking this into account, this phase of the menstrual cycle was used for the study, but it is worth noting that the ideal would be in the secretory phase, and this may have been a factor influencing the result. Likewise, there is a study by Marin *et al*., 2022, in which the authors found no significant difference even when performing the biopsy in the secretory phase of the menstrual cycle.

## CONCLUSION

The expression of *HOXA10* and *HOXA11* during the proliferative phase is not significantly different between infertile women with endometritis and infertile women without endometritis. Translational studies with a larger number of patients should be performed.
